# Medical accessibility and underreporting of occupational diseases: effect of travel distance and travel time

**DOI:** 10.3389/fresc.2025.1545460

**Published:** 2025-04-07

**Authors:** Ping Hui Chen, Po-Ching Chu, Ching-Chun Huang, Chi-Hsien Chen, Yue Leon Guo, Ta-Chen Su, Pau-Chung Chen

**Affiliations:** ^1^ Department of Environmental and Occupational Medicine, National Taiwan University Hospital Hsin-Chu Branch, Hsinchu, Taiwan; ^2^ Department of Environmental and Occupational Medicine, National Taiwan University Hospital, Taipei, Taiwan; ^3^ Department of Public Health, College of Public Health, National Taiwan University, Taipei, Taiwan; ^4^ Institute of Environmental and Occupational Health Sciences, College of Public Health, National Taiwan University, Taipei, Taiwan; ^5^ National Institute of Environmental Medicine, National Health Research Institutes, Zhunan, Taiwan

**Keywords:** occupational disease, GIS, geographic information system, medical accessibility, underreporting, IRR, incidence rate ratio, occupational health centers

## Abstract

**Objectives:**

Underreporting of occupational diseases (ODs) could be attributed to poor medical accessibility, which is rarely discussed previously. Our cross-sectional study aims to evaluate how OD reporting is impeded by long travel distance/time (TD/TT) to the nearest major occupational medicine clinics.

**Methods:**

Using data from the Network of Occupational Diseases and Injuries Service (NODIS), Taiwan's OD surveillance system, and the annual Manpower Survey from 2008 to 2018, we calculate each district's incidence rate of ODs (IROD) and expected IROD based on industries and job titles. Each town's TD/TT to the nearest major occupational medicine clinics is estimated by Google Maps’ Distance Matrix API. The quasi-Poisson regression model is used to investigate the effect of TD and TT on IROD, while industries and job titles are adjusted by offsetting expected IROD. A subgroup analysis is then carried out to check the effect of employment status, sickness absence, and reporting years.

**Results:**

A total of 3,420 cases of definite ODs are included in our study. Using the quasi-Poisson regression model, after adjusting industry types and job titles, TD and TT have a significant effect on IROD. As TD/TT increases by 10 km/10 min, IROD decreases by 10.90%/10.73%. It is estimated that ∼200 OD cases per year or 40% of ODs are therefore underreported. In the subgroup analysis, only mildly sick workers are still significantly affected by TD and TT.

**Conclusions:**

Our study shows how poor medical accessibility leads to underreporting, especially for mildly sick cases, and up to 40% of ODs could be underreported. Using this method, we can evaluate the cost-effectiveness of adding reporting hospitals in areas with poor medical accessibility.

## Introduction

In most countries with worker compensation insurance, the authority concerned could utilize insurance data to monitor the incidence of occupational diseases (ODs), as an important indicator of occupational safety and health (1–5). However, workers are encountered with several filters, which eventually lead to underdiagnosis and underreporting of occupational diseases (5, 6). Thus, some countries establish reporting systems encouraging occupational physicians and other physicians to diagnose and report occupational diseases. Because most physicians have inadequate training or few incentives to diagnose and report occupational diseases (7–9), many countries adopted a model of occupational health centers composed of occupational physicians to maintain these reporting systems (10), including France (11), the United Kingdom (12), and Taiwan (13). However, medical accessibility of these occupational health centers could be a problem leading to underreporting.

Medical accessibility is an important factor influencing patients’ medical-seeking behavior. There are numerous indicators of medical accessibility, and traveling distance (TD)/time is one of the most important indicators, which affects all kinds of patients’ medical-seeking behaviors. Several previous studies used the open-source modules ArcView and AccessMod to estimate the shortest path and travel time to the nearest health facilities (14–19), as well as other studies using Google Maps API to carry out the estimation (20). Patients faced with long traveling distance/time are less likely to seek treatment in time (21–25) and more likely to have poor prognosis due to delayed treatment (16, 26). Thus, for policymakers, it is crucial to identify areas lacking accessible healthcare and evaluate the cost-effectiveness of adding additional healthcare facilities (27).

Although Taiwan's healthcare system is well known for its great medical accessibility due to National Health Insurance and dense population, medical accessibility is still an important consideration for patients seeking healthcare. In the previous study, it is found that 86.4% of clinic services and 77.3% of emergency room hospitalization services occur in patients’ residences and nearby administrative districts, and the average weighted travel distance for clinic services and emergency room hospitalization services is 17.68 and 28.78 km, respectively. It is concluded that the average weighted travel distance is a major factor determining patients’ medical-seeking behavior, especially for clinic service (28).

Considering the lack of time for workers to visit clinics and the scarcity of occupational physicians and occupational medicine clinics, it is expected that medical accessibility would be an important consideration for workers seeking diagnosis of occupational diseases. Thus, it 's not surprising that poor medical accessibility leads to underdiagnosis and underreporting of occupational diseases. However, only one French study has discussed it previously.

In France, the French National OD Surveillance and Prevention Network (RNV3P) is an important reporting system, composed of 31 occupational disease clinics (OD clinics), which act as occupational health centers. In the previous study, by calculating the ratio between the numbers of observed occupational diseases in RNV3P and the numbers of expected occupational diseases, which is estimated based on the number of employees for each activity sector and the average rate of occupational diseases by activity sector in each administrative districts, it is found that the rate of observations (incidence of occupational diseases) decreases with increasing distance from the OD clinics (29).

However, this study did not quantify the effect of medical accessibility, which largely limits its practical implications. For policymakers, quantitative analysis is necessary for identifying areas that lack occupational medicine clinics the most and for carrying out cost-effectiveness evaluation to determine whether establishing a new occupational health center is worth it or not. All these research gaps call for further study to quantify the effect of medical accessibility and demonstrate its practical implications.

In Taiwan, underdiagnosis and underreporting of occupational diseases have long been a serious unsolved problem, which troubles workers, occupational physicians, and policymakers a lot (5, 13). Compared with other countries, the incidence of occupational diseases in Taiwan is extremely low, which is explained by underdiagnosis and underreporting of occupational diseases (5, 13).

To solve the problem of underreporting, the authority concerned has established the Network of Occupational Diseases and Injuries Service (NODIS) since 2008, which is composed of major reporting hospitals and numerous nearby minor reporters coordinated by these major reporting hospitals. Supported by major reporting hospitals, which act as occupational health centers, the incidence of occupational diseases and outpatient visits to occupational medicine clinics have greatly increased since the establishment of NODIS in 2007 (5, 13, 30). Although the number of major reporting hospitals has increased over time, some areas are still remote from the nearest major reporting hospital and lack occupational medicine clinics.

Considering the distribution and scarcity of major reporting hospitals and their importance in the NODIS system, traveling distance and traveling time (TT) to the nearest major reporting hospitals are better indicators of medical accessibility, compared with other measures, such as the number of occupational physicians (many occupational physicians do not provide clinical services in major reporting hospitals), availability of occupational medicine clinics (only major reporting hospitals routinely provide well-functioned clinical services), or density of major reporting hospitals (not applicable for most districts with no major reporting hospitals).

Thus, our study aims to investigate and quantify the effect of medical accessibility of occupational medicine clinics on the incidence rate of occupational disease, by using traveling distance and traveling time as indicators. Our study could fill the aforementioned research gaps about quantitative analysis and its practical implications. We could estimate not only how many cases of occupational diseases are underreported due to poor medical accessibility but also how many cases of occupational diseases could be reported additionally if one additional major reporting hospital is established in areas lacking occupational medicine clinics.

## Methods

### Study population and data sources

In our cross-sectional study, we extract cases of occupational diseases from the Network of Occupational Diseases and Injuries Service (NODIS), which has been the main occupational diseases reporting system in Taiwan since 2008 till now. It is composed of nine major reporting hospitals and numerous nearby minor reporters coordinated by these major reporting hospitals. Occupational physicians in these healthcare facilities would report suspected cases of occupational diseases and usually visit occupational medicine clinics to claim benefits from worker compensation insurance. Three occupational physicians appointed by the NODIS would determine each case's work-relatedness and categorize them into probable (>50% chance), possible (<50% chance), and non-related (non-qualified cases) based on their consensus.

For each reported case, the occupational physicians are required to report many variables, including year, administrative district of workplace, job titles, industries, sick leave records, employment status, disease diagnosis, occupational exposure, temporality, epidemiological evidence, and other etiologies. These data are mainly based on comprehensive history taking at the clinic, which could also be supported by additional information provided by patients, such as certificates of their worker compensation insurance, medical charts, attendance records, and work photos/videos. If necessary, we could also arrange worksite visits and workplace environmental monitoring to obtain more information.

Among these variables, industries are categorized into 19 categories (A–S) in the Standard Industrial Classification System, while job titles are categorized into 10 categories (0–9) in the Standard Occupational Classification System. These two systems are both national classification systems widely used in Taiwan. Administrative districts of workplace are categorized into 368 tertiary administrative divisions, yet some downtown small tertiary divisions would be grouped together due to data limitations. Disease diagnoses are categorized by the International Classification of Disease and Related Health Problems, 9th Edition (ICD-9), and worker compensation insurance's list of occupational diseases. To avoid misclassification of NODIS data, we reclassify disease diagnoses for all NODIS cases one by one, based on the contents reported by occupational physicians. Sick leave records and employment status are true–false questions self-reported by patients, while disease diagnosis, occupational exposure, temporality, epidemiological evidence, and other etiologies are variables answered in text by occupational physicians.

To calculate the incidence rate of occupational diseases in each administrative district, we further extract data from the Manpower Survey, which is a yearly national manpower survey conducted by the Taiwanese Government's Directorate-General of Budget, Accounting and Statistics (31). Using stratified two-stage random sampling methods, certain families are identified as sample families. Appointed interviewers would interview them by visiting or telephone and record detailed employment status in these families. The number of workers and their characteristics, including administrative district of workplace, year, job titles, and industries, could then be estimated by multiplying its weighting.

For better understanding, Table 1 lists all the variables included in our study, with their data source and description. Because we only use the required reported variables for analysis in our study, there is no issue of missing data.

**Table 1:** Variables included in our study.

Data source	Variables	Description
NODIS (reported cases of occupational diseases)	Administrative district of workplace	368 Taiwanese government's tertiary administrative divisions
	Year	Reported year
	Job titles	10 categories in Taiwan's standard occupational classification system
	Industries	19 categories in Taiwan's standard industrial classification system
	Sick leave records	True–false questions self-reported by patients
	Employment status	
	Work-relatedness	Evaluated (probable/possible/non-related) by three occupational physicians appointed by NODIS
	Disease diagnosis	ICD-9/worker compensation insurance list of occupation diseases
	Disease diagnosis, occupational exposure, temporality, epidemiological evidence, other etiologies	Texts answered by occupational physicians
Manpower Survey (working population)	Administrative district of workplace	368 Taiwanese government's tertiary administrative divisions
	Year	Survey year
	Job titles	10 categories in Taiwan's standard occupational classification system
	Industries	19 categories in Taiwan's standard industrial classification system

NODIS, Network of Occupational Diseases and Injuries Service; ICD-9, International Classification of Disease and Related Health Problems, 9th Edition.

### Ethics approval

For the abovementioned data sources, including the NODIS and Manpower Survey, only routinely collected non-identifiable data are collected in advance. Thus, no ethics approval was obtained for our study, because we only analyzed these routinely collected non-identifiable data afterward.

### Inclusion and exclusion criteria

Using data from the NODIS and Manpower Survey, we can calculate the incidence rate of occupational diseases in each administrative district from January 1, 2008, to December 31, 2018. All probable cases in the NODIS are included in our study, while certain types of occupational diseases are excluded:

• Cases of abnormal health check-ups and occupational injury are excluded due to not meeting the definition of occupational disease.
• Cases of skin diseases and decompression syndrome are excluded, because these cases usually visit dermatology and pulmonology clinics and are then reported by dermatologists and pulmonologists, while most cases of other occupational diseases visit occupational medicine clinics and are reported by occupational physicians.
• Cases of pneumoconiosis are excluded, because we cannot distinguish them from cases of abnormal health check-ups.

### Estimation of traveling distance and traveling time

We use Google Maps Distance Matrix API to estimate the traveling distance (TD) and traveling time (TT) from workplace to the nearest major reporting hospitals. The detailed setting of Google Maps Distance Matrix API is as follows:

• Origin: district office of workplace's administrative district.
• Destination: nearest major reporting hospitals.
• Traveling mode: driving.
• Departure time: not specified. The result would be based on average time-independent traffic conditions.

Taking real-world road network and traffic conditions into consideration, the Google Maps Distance Matrix API can best estimate actual traveling distance and traveling time from a worker's workplace to the nearest major reporting hospitals.

**Figure 1:**
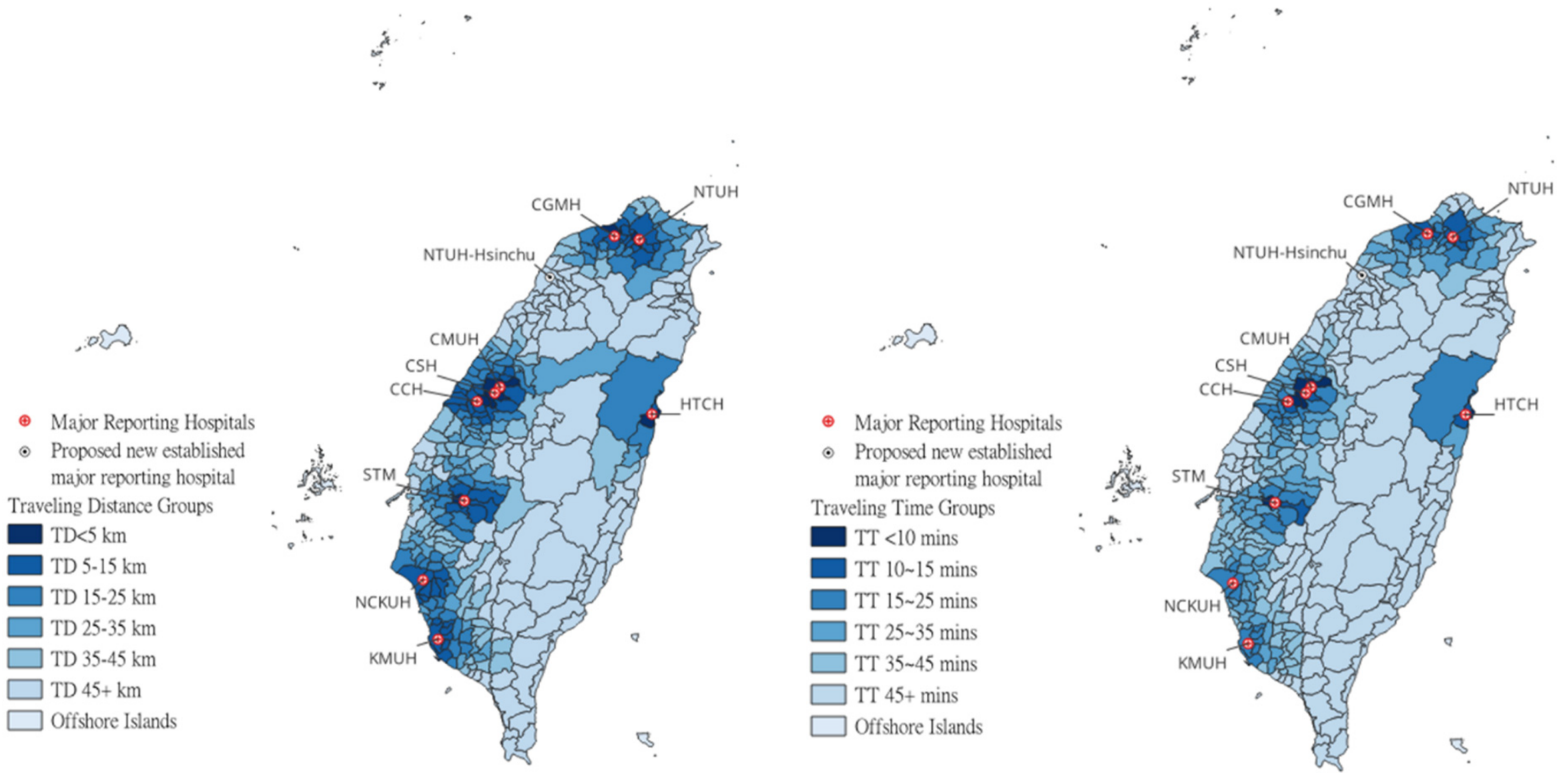
Locations of nine major reporting hospitals and NTUH Hsinchu and each district's TD/TT groups.

For a better understanding of the spatial distribution of major reporting hospitals and their nearby administrative districts’ medical accessibility, Figure 1 labels these nine major reporting hospitals and TD/TT groups of each district on the map of Taiwan. We also label the location of the National Taiwan University Hospital Hsinchu branch (NTUH Hsinchu), which would be later used as an example, to show what could be expected if one additional major reporting hospital is established in an area with poor medical accessibility.

### Confounders

Among all the confounders related to the incidence rate of occupational diseases, job titles and industries are the most important ones. To adjust different workforce composition, we calculate the expected incidence of occupational diseases for each administrative district, based on the incidence rate of occupational diseases by each job title and industry in districts nearest to major reporting hospitals (TD < 5 km or TT < 10 mins) and district's proportion of workers by each job title and industry. The aforementioned method has also been adopted by previous studies for calculating the expected incidence of occupational diseases (29):

$$\mathrm{IE}=\;\overset{X=J,\;Y=s}{\underset{X=A,\;Y=a}{\sum }}\;I(X,Y)*P(X,Y)$$

IE: expected incidence rate of occupational disease

*I*: incidence rate of occupational disease by each job title and industry in districts nearest to major reporting hospitals.

*P*: proportion of workers by each job title and industry

*X*: job titles, *A*–*J*

*Y*: industries, *a*–*s*

After adding up the multiplied incidence rate and proportion of workers, we can get the expected incidence rate of occupational disease for each administrative district. By putting this variable into our regression model, we can adjust the effect of different workforce composition.

For better understanding, if workers in one administrative district are composed of 40% of *X* workers (industry *A* and job title *a*) and 60% of *Y* workers (industry *B* and job title *b*), while the incidence rate of occupational diseases in districts nearest to major reporting hospitals (TD < 5 km or TT < 10 mins) for *X* and *Y* workers are 100 and 50 cases per million workers, respectively, the expected incidence rate of occupational disease in this administrative district would be 70 (0.4 × 100 + 0.6 × 50) cases per million workers. To adjust the effect of job title and industry, we would then put this variable into a quasi-Poisson regression model as an offset. If one administrative district's incidence rate of occupational diseases is higher just because of its workforce composition, it would then be adjusted by its expected incidence rate.

### Statistical analysis

Administrative districts would be divided into different groups by TD and TT. By TD, these districts are divided into TD < 5 km, TD 5–15 km, TD 15–25 km, TD 25–35 km, TD 35–45 km, TD >45 km, and off-shore islands. By TT, these districts are divided into TT <10 min, TT 10–15 min, TT 15–25 min, TT 25–35 min, TT 35–45 min, TT > 45 min, and off-shore islands.

To investigate the effect of TD and TT on the incidence rate of occupational diseases, we first carry out descriptive analysis and present cases of occupational diseases, worker-years, and incidence rate of occupational diseases for each TD and TT group.

We further use the quasi-Poisson regression model to investigate the effect of TD and TT on the incidence rate of occupational diseases, while the expected incidence rate of occupational disease is set as offset, to adjust the confounding effect of job titles and industries. A similar method has also been adopted by a previous study for adjusting confounders (29). In the quasi-Poisson regression model, the relationship among TD/TT, incidence rate of occupational diseases, and expected incidence rate of occupational disease is as follows:

lnI=α+β*X+lnIE

$$\gg \ln\;I_{1}/I_{0}=\;\beta *(X_{1}-X_{0})$$

$$\gg I_{1}/I_{0}=e^{\beta *X_{1}-X_{0}}$$

IE: expected incidence rate of occupational disease.

*I*: incidence rate of occupational disease.

*X*: TD/TT, groups of TD/TT.

*β*: coefficient of *X*, *e^β^* represents the incidence rate ratio (IRR).

*α*: constant.

In this quasi-Poisson regression model, *e^β^* is our main result, which represents the incidence rate ratio (IRR), compared to the nearest district. If IRR and its 95% confidence interval (CI) are <1.0, it means the incidence rate of occupational diseases in the district is significantly less than the nearest district and the value of IRR also represents the level of underreporting.

We would perform all the statistical analysis by the software R (version 4.3.1). The statistical significance level was set at a two-sided *p*-value of <0.05.

### Subgroup analysis

We then carry out subgroup analysis to investigate the effect workers’ sick leave records, employment status, and reported year. Cases of occupational diseases would be divided into taking sick leaves/no sick leaves, unemployed/employed, and earlier cases (2008–2013)/later cases (2014–2018). We then use the quasi-Poisson regression model again to check whether IRR would change or not in different subgroups.

### Estimation of underreported cases of occupational diseases

Furthermore, by simple calculation, we could use our model to estimate the number of underreported cases of occupational diseases attributable to poor medical accessibility annually and how many cases of occupational diseases could be reported additionally if one additional major reporting hospital is established, by the example of National Taiwan University Hospital Hsinchu branch.

## Results

From January 1, 2008, to December 31, 2018, there were 23,426 cases of occupational diseases reported to the NODIS. After excluding 14,936 cases of abnormal health check-ups, pneumoconiosis, occupational injury, skin diseases, and decompression syndrome and 4,383/687 possible/non-related cases, there are 3,420 probable cases included in our study. Among these 3,420 cases, 2,401 (70%) cases are reported by major reporting hospitals, while other 999 (30%) cases are reported by nearby minor coordinated reporters.

Among these 3,420 cases, 143 cases had no fixed workplace, and 4 cases came from off-shore islands. As Table 2 shows, the incidence rate of occupational diseases is 26.54 per million worker-years for all districts. Generally, the incidence rate declines as TD and TT increase, while the lowest incidence rate is 9.31 per million worker-years for off-shore islands.

**Table 2:** Incidence rate and incidence rate ratio of occupational diseases by TD/TT groups during 2008–2018.

TD/TT groups	Cases	Worker-years	Incidence rate per million	Incidence rate ratio	95% CI
Total	3,420	123,487,315	26.54		
Traveling distance					
TD <5 km	652	15,671,173	41.61	100%	100%
TD 5–15 km	1,425	56,912,790	25.04	61.60%	49.94%–75.98%
TD 15–25 km	607	20,098,044	30.20	60.42%	47.02%–77.64%
TD 25–35 km	222	9,248,727	24.00	49.97%	35.37%–70.59%
TD 35–45 km	134	6,723,588	19.93	44.99%	29.51%–68.59%
TD >45 km	233	14,403,324	16.18	31.92%	22.47%–45.34%
Off-shore islands	4	429,670	9.31	15.90%	1.71%–147.82%
Traveling time					
TT <10 mins	490	11,568,198	42.36	100%	100%
TT 10–15 mins	224	5,868,507	38.17	69.06%	48.07%–99.22%
TT 15∼25 mins	1,324	54,399,581	24.48	56.96%	44.93%–72.19%
TT 25–35 mins	687	24,890,756	27.60	52.59%	40.32%–68.60%
TT 35–45 mins	300	11,476,910	26.14	52.30%	37.63%–72.70%
TT >45 mins	248	14,853,694	16.35	30.12%	21.01%–43.18%
Off-shore islands	4	429,670	9.31	14.71%	1.54%–140.24%
No fixed workplace	143				

TD, traveling distance; TT, traveling time; CI, confidence interval.

To adjust the confounding effect of job titles and industries, we first carry out the quasi-Poisson regression model by groups of TD and TT, while the expected incidence rate of occupational disease is set as an offset. As Table 2 shows, compared with nearest districts with TD < 5 km or TT < 10 min, IRR significantly declines as TD and TT increase, and there is an obvious dose–response relationship. For districts whose TD is >45 km or TT is >45 min, IRR could be as low as 31.92% and 30.12%, which means 68.08% and 69.88% cases of occupational diseases are underreported due to long TD and TT, respectively, compared with nearest districts like TD < 5 km or TT < 10 min.

We then carry out the quasi-Poisson regression model with TD and TT as continuous variables. As Table 3 shows, IRR significantly declines as TD and TT increase. When TD and TT increase by 10 km and 10 min, IRRs significantly reduce to 89.10% and 89.27%, which means 10.90% and 10.73% of cases of occupational diseases are underreported due to increased TD and TT, respectively.

**Table 3:** Incidence rate ratio by TD/TT in different subgroups during 2008–2018.

Subgroups	Estimate	SD	IRR	95% CI
Traveling distance			(Per 10 km)	(Per 10 km)
All	−0.013	0.0025	89.10%	84.84%–93.56%
Sickness absence (−)	−0.016	0.0033	85.11%	79.78%–90.80%
Sickness absence (+)	−0.005	0.0037	94.82%	88.16%–101.97%
Keep jobs	−0.014	0.0025	86.57%	82.40%–90.96%
Lose jobs	−0.001	0.0045	99.06%	90.77%–108.11%
Earlier (2008–2013)	−0.017	0.0049	84.48%	76.74%–93.01%
Later (2014–2018)	−0.012	0.0031	88.78%	83.59%–94.29%
Traveling time			(per 10 min)	(per 10 min)
All	−0.013	0.0029	89.27%	84.48%–94.33%
Sickness absence (−)	−0.018	0.0039	83.31%	77.17%–89.93%
Sickness absence (+)	−0.003	0.0038	96.96%	90.05%–104.41%
Keep jobs	−0.015	0.0029	85.72%	80.96%–90.76%
Lose jobs	0.003	0.0043	102.98%	94.74%–111.95%
Earlier (2008–2013)	−0.018	0.0056	83.19%	74.50%–92.90%
Later (2014–2018)	−0.012	0.0035	89.12%	83.24%–95.41%

TD, traveling distance; TT, traveling time; SD, standard deviation; IRR, incidence rate ratio; CI, confidence interval; km, kilometer; min, minute.

Furthermore, we also carry out subgroup analysis to investigate IRR in different subgroups, such as having sickness absence or not, keeping or losing jobs, and earlier or later cases. As Table 3 shows, for those severely sick workers who ever have sickness absence or lose their jobs, the effects of TD and TT are no longer significant. Meanwhile, for those mildly sick workers, the effects of TD and TT are still significant and even larger. When we separate these cases into earlier cases (2008–2013) and later cases (2014–2018), the effects of TD and TT are all still significant, yet slightly larger for earlier cases.

Based on the aforementioned results, we already have each district's worker-years, incidence rate of occupational diseases, and incidence rate ratio (also known as the level of underreporting) by their TD/TT groups. Using these variables, we could easily estimate how many cases of occupational diseases are underreported due to poor medical accessibility. It is estimated that the incidence rate could increase from 26.54 to 44.03 or 47.28 per million worker-years if the effect of TD/TT is removed, which means 39.73%/43.87% or 196.36/232.82 cases of occupational diseases are underreported due to long TD or TT annually.

Among those districts with long TD/TT, Hsinchu and Miaoli are areas where numerous workers work in high-risk manufacturing industries. Using TD/TT groups used in our study as an indicator of medical accessibility, we could identify areas lacking occupational medicine clinics the most. In Hsinchu and Miaoli, almost all workers (94.95%/87.71%) work in districts with TD > 45 km or TT > 45 min, while about half (54.82%/49.11%) workers work in these districts come from Hsinchu and Miaoli. In fact, among the aforementioned 200 underreported cases annually, ∼14.5 cases come from Hsinchu and Miaoli. Thus, apparently, there is an urgent need to promote medical accessibility of occupational medicine clinics.

If we establish one additional major reporting hospital at the site of the National Taiwan University Hospital Hsinchu branch, medical accessibility of occupational medicine clinics would be promoted greatly, as TD/TT would be greatly reduced. As Table 4 shows, after one additional major reporting hospital is established, districts whose TD > 45 km, TT > 45 min, or TT = 35–45 min are reclassified into new groups with decreased TD/TT, which makes their IRR and expected incidence rate increase at the same time. It is estimated that the incidence rate could increase from 9.35/10.35 to 21.94/22.91 per million worker-years, which means an additional 43.00%/39.64% or 8.70/7.72 cases of occupational diseases could be reported after one additional major reporting hospital is established.

**Table 4:** Expected changes if one additional major reporting hospital is established.

Changes in TD/TT groups	Worker-years	Incidence rate per million	Original IRR	Improved IRR	Improved incidence rate	Yearly reported cases
Changes in TD groups						
45+ km → <5 km	4,448,979	7.42	31.92%	100%	23.24	6.40
45+ km → 5–15 km	442,446	15.82	31.92%	61.60%	30.53	0.59
45+ km → 15–25 km	1,246,133	7.22	31.92%	60.42%	13.67	0.73
45+ km → 25–35 km	695,681	15.81	31.92%	49.97%	24.75	0.57
45+ km → 35–45 km	764,068	14.40	31.92%	44.99%	20.29	0.41
Total	7,597,307	9.35	31.92%	74.92%	21.94	8.70
Changes in TT groups						
45+ min → <10 min	3,537,856	6.50	30.12%	100%	21.58	4.85
45+ min → 10–15 min	911,123	10.98	30.12%	69.06%	25.16	1.18
45+ min → 15–25 min	876,527	5.70	30.12%	56.96%	10.79	0.41
45+ min → 25–35 min	630,789	12.68	30.12%	52.59%	22.14	0.54
45+ min → 35–45 min	803,792	13.69	30.12%	52.30%	23.76	0.74
35–45 min → 25–35 min	827,406	15.71	52.30%	52.59%	15.80	0.01
Total	6,760,087	10.35	32.70%	72.34%	22.91	7.72

TD, traveling distance; TT, traveling time; IRR, incidence rate ratio; CI, confidence interval; km, kilometer; min, minute.

## Discussion

Our study shows that long traveling distance and traveling time, as indicators of medical accessibility, leads to a significantly lower incidence rate of occupational diseases, which is consistent with a previous French RNV3P study (29). After adjusting different workforce composition of job titles and industries, there is an obvious dose–response relationship between IRR and TD/TT. When TD and TT increase by 10 km and 10 min, IRR significantly reduces to 89.10% and 89.27%, which means 10.90% and 10.73% cases of occupational diseases would be underreported due to increased TD/TT, respectively, compared with the nearest districts.

Furthermore, in our subgroup analysis, our study shows that the effect of TD/TT is associated with incentives and only applies to workers who have no sickness absence or keep their jobs. These mildly sick workers usually have weaker incentives to visit occupational medicine clinics and undergo work-relatedness evaluation. Thus, their medical-seeking behavior would be easily discouraged by long traveling distance and traveling time. In comparison, severely sick usually have stronger incentives and are less discouraged by long traveling distance and traveling time.

In subgroup analysis, we also found that the effect of TD/TT became slightly smaller during a later period (2014–2018). This temporal change implies that the NODIS continues to enhance its medical accessibility of occupational medicine clinics as time goes by. From 2008 to 2015, the number of funded weekly clinics increased from 142 to 225, while the number of first outpatient visits increased from 1,777 to 7,374.

Based on the aforementioned quantitative analysis, it is estimated that 39.73%/43.87% or 196.36/232.82 cases of occupational diseases are underreported due to long TD or TT annually. In addition, using TD/TT groups as an indicator of medical accessibility, we could easily identify Hsinchu and Miaoli as areas lacking occupational medicine clinics the most. If we establish one additional major reporting hospital at the site of the National Taiwan University Hospital Hsinchu branch, the incidence rate could increase from 9.35/10.35 to 21.94/22.91 per million worker-years in Hsinchu and Miaoli, which means an additional 43.00%/39.64% or 8.70/7.72 cases of occupational diseases could be reported.

This study still has several limitations. Firstly, the NODIS is a database whose accuracy largely depends on reporters’ reporting quality due to lack of enough afterward validation, and variables such as workplace, job titles, industries, sick leave records, and employment status could be misclassified. However, the NODIS is the best available and reliable data source in Taiwan. We could still evaluate the magnitude of these misclassifications by checking whether the results of our analysis are reasonable or not. Also, the Manpower Survey is conducted by yearly stratified sampling, based on household registration. There would be inevitable sampling errors, especially in districts with few samples. However, the Manpower Survey is also the best available and reliable data source in Taiwan. We could also validate its accuracy by referring to other manpower survey data.

Secondly, there are still other unadjusted confounders, such as age/tenure, gender, and corporation sizes, which also have effects on the incidence rate of occupational diseases. However, these adjustments may be not feasible, because the number of workers is not large enough for further division in some districts, and some variables in the Manpower Survey may not be reported in the NODIS database. Afterall, we still adjust different workforce composition of job titles and industries, which may be the most important confounders.

Thirdly, there are still several limitations in the estimation of traveling distance and time. In Google Maps Distance Matrix API, origin, destination, traveling mode, and departure time are variables used for estimation. As for origin, workers may depart from their residence or workplace. However, due to lack of workers’ residence or actual address of their address in the NODIS database, we could only set their origin as district office of their workplace's administrative district, which is the best available data that we have. As for traveling mode, there are ways other than driving for workers visiting occupational medicine clinics, such as public transportation. However, considering underdeveloped public transportation in many districts and inaccurate or incomplete data on public transportation in Google Maps Distance Matrix API, driving mode is still the most preferrable setting for traveling mode. At last, because the estimation was carried out by Google Maps Distance Matrix API in 2019–2020, the estimation would still be different from the actual scenario during 2008–2018. It is expected that traveling distance and traveling time may be underestimated, yet these estimations are believed to be the best available, and there would only be limited bias because the estimation is based on average time-independent traffic conditions.

Based on our study's findings, we have advised authorities concerned about establishing more major reporting hospitals, especially in areas with numerous workers and poor medical accessibility of occupational medicine clinics, such as Hsinchu and Miaoli. After worker compensation insurance and the NODIS system were reformed during 2022–2023, more major reporting hospitals were established, including the National Taiwan University Hospital Hsinchu branch. Thus, our study calls for further studies in the near future to validate whether our estimation meets the actual scenario or not and how incidence rates of occupational diseases would change in the NODIS system, especially in areas where new major reporting hospitals are established. In addition, further studies may also overcome some aforementioned limitations with some reporting variables and mechanisms renewed.

## Conclusions

Our study shows that long traveling distance and traveling time, as indicators of medical accessibility, leads to underreporting of occupational diseases, especially for mildly sick cases. Approximately 10% of cases of occupational diseases could be underreported as traveling distance or traveling time increases by 10 km or 10 min, and 40% of ODs could thus be underreported.

Using this method, we can evaluate the cost-effectiveness of adding reporting hospitals in areas with poor medical accessibility by estimating how many cases of occupational diseases could be reported additionally.

## Data Availability

The raw data supporting the conclusions of this article will be made available by the authors, without undue reservation.
